# Histopathology-Paired Clinical Improvement Following Topical *Rosa damascena*-Derived Exosomes in Long-Standing Refractory Male Genital Lichen Planus: A Single-Patient Case Report

**DOI:** 10.3390/ph19071010

**Published:** 2026-06-29

**Authors:** Michał Kaniowski, Lidia Majewska, Zdzisław Woźniak, Ewa Kaniowska, Karolina Dorosz

**Affiliations:** 1 Kaniowscy Clinic, Private Practice, ul. Barycka 1/3, 50-325 Wrocław, Poland; biuro@kaniowscy.pl (M.K.); e.kaniowska@kaniowscy.pl (E.K.); 2 ESME Clinic, Private Practice, ul. Lwowska 1/u16, 30-548 Kraków, Poland; 3 Cosmetology Department, University of Kalisz, Plac Wojciecha Bogusławskiego 2, 62-800 Kalisz, Poland; zwozniak9@wp.pl; 4 Biological Sciences Division, University of Chicago, Chicago, IL 60637, USA; kdorosz@uchicago.edu

**Keywords:** lichen planus, genital dermatosis, plant-derived exosomes, *Rosa damascena*, case report, histopathology

## Abstract

Lichen planus (LP) is a chronic T-cell-mediated interface dermatitis. Genital involvement is frequently refractory to topical corticosteroids and calcineurin inhibitors and may lead to fibrosis, architectural distortion, and substantial impairment in quality of life. We report the case of a 39-year-old male with a 15-year history of biopsy-confirmed genital LP unresponsive to high-potency topical corticosteroids and tacrolimus 0.1%, who received topical *Rosa damascena* stem cell-derived exosomes (RSCEs) at biweekly sessions for four months. Each session combined 2 mL of in-office application with superficial microneedling in hyperkeratotic areas, followed by 3 mL of the same-day home application. No concomitant topical corticosteroid or calcineurin inhibitor was used during the treatment period. Paired pre- and post-treatment 4 mm punch biopsies were obtained from the same anatomical region, processed using identical protocols, stained with hematoxylin and eosin, and reviewed by a board-certified dermatopathologist. After four months, we observed clinical resolution of pruritus and fissuring, progressive desquamation of hyperkeratotic plaques, and improved tissue elasticity. The post-treatment biopsy showed reduced hyperkeratosis and hypergranulosis, attenuation of the band-like lymphohistiocytic infiltrate, partial restoration of the dermoepidermal interface, and reduced basal vacuolar degeneration relative to baseline. No dysplastic changes or treatment-related adverse events were observed. These observations are based on a single uncontrolled case and cannot establish causality, isolate the contribution of microneedling, or demonstrate disease modification beyond the descriptive level. Histological assessment was qualitative; no semi-quantitative or immunohistochemical analysis was performed. The exosome preparation was used as a standardized commercial product and was not independently characterized in our laboratory. The findings are intended solely as hypothesis-generating. Independent characterization of the exosome preparation, immunohistochemical and ideally transcriptomic profiling of paired tissue, and prospective controlled studies are required before any therapeutic claim can be supported.

## 1. Introduction

Lichen planus (LP) is a chronic, immune-mediated inflammatory dermatosis affecting approximately 0.5–1% of the general population and encompassing cutaneous, mucosal, and appendageal variants [1]. Male genital involvement represents a diagnostically challenging and often underrecognized manifestation that may present as hyperkeratotic, erythematous, annular, or atrophic plaques of the glans penis and foreskin [2,3,4,5]. Long-standing genital LP may result in fibrosis, architectural distortion, sexual dysfunction, and meaningful impairment of quality of life [6,7], and chronic mucosal disease carries a small but documented risk of malignant transformation [1].

At the molecular level, LP is a prototypical interface dermatitis driven by a CD8+ T-cell response against basal keratinocytes within a Th1-skewed cytokine environment, including IFN-γ, TNF-α, and IL-6, with downstream JAK/STAT activation [1]. Histopathologically, this manifests as basal vacuolar degeneration, a band-like lymphohistiocytic infiltrate, and disruption of the basement membrane zone.

Management remains predominantly suppressive. High-potency topical corticosteroids and calcineurin inhibitors are first-line treatments, but in long-standing genital disease, they frequently fail to achieve durable structural normalization [8,9]. Refractory cases may benefit from systemic immunomodulators or, more recently, JAK inhibitors [10], although these act through pathway suppression rather than tissue remodeling and carry the risk profile of systemic immunosuppression.

Extracellular vesicles (EVs), in particular exosomes, are lipid-bilayer–enclosed nanovesicles (≈30–200 nm) that mediate intercellular transfer of microRNAs, proteins, and lipids and have shown immunomodulatory and regenerative activity in preclinical models [11,12]. Plant-derived exosome-like nanoparticles (PDENs) have attracted attention because of their scalability, low immunogenicity, and bioactive cargo [11,12]. *Rosa damascena* stem cell-derived exosomes (RSCEs) are a commercial PDEN preparation manufactured using the ExoSCRT mass-culture and purification platform. Manufacturer-provided characterization and one independent compositional study describe an EV size distribution of approximately 90–200 nm and a cargo containing microRNAs (including Let-7 family members), proteins, and lipid species, with a reduction in IL-6 release in activated macrophages and stimulation of fibroblast proliferation in vitro [13].

Building on these in vitro characterization data, preliminary clinical experience with RSCEs in dermatology has begun to accumulate. A retrospective case-series with long-term outcome assessment reported favorable responses to standardized RSCE preparation across a range of cutaneous wound-healing and scar-remodeling indications, with sustained clinical improvement and no significant adverse events on extended follow-up [14]. More broadly, plant-derived extracellular vesicles-including RSCEs—have been positioned in recent reviews as an emerging therapeutic category in dermatology, with putative immunomodulatory, anti-inflammatory, and pro-regenerative properties of potential relevance to chronic inflammatory and fibrosing cutaneous disorders [15]. To our knowledge, however, no prior report has examined their application in genital lichen planus.

Within this context, we report a histopathology-paired clinical observation in a single patient with refractory male genital LP. We present this case as hypothesis-generating; we do not propose RSCEs as an established treatment; the analysis presented is descriptive, and the brief mechanistic context in the discussion is explicitly speculative.

## 2. Case Presentation

A 39-year-old male presented with a 15-year history of chronic inflammatory dermatosis affecting the glans penis and foreskin, previously labeled clinically as lichen sclerosus and treated intermittently with high-potency topical corticosteroids and tacrolimus 0.1%. Misclassification between lichen planus and lichen sclerosus at this site is well documented because of overlapping clinical and histological features [16].

**Figure 1:**
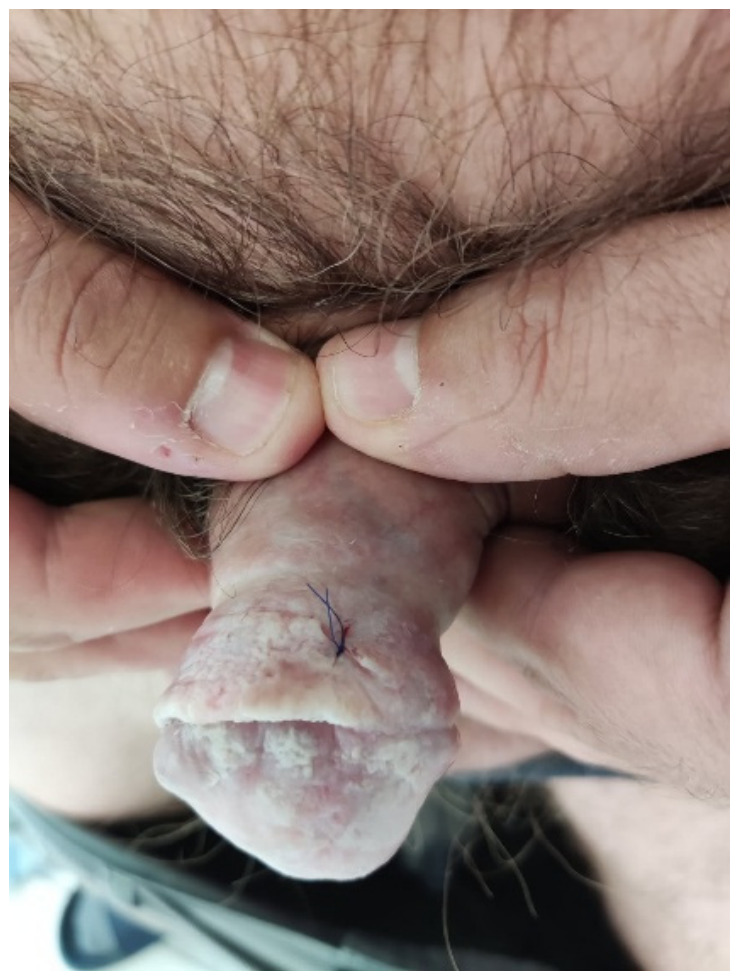
Clinical presentation before treatment, showing marked hyperkeratosis and fibrotic tightening of the foreskin.

**Figure 2:**
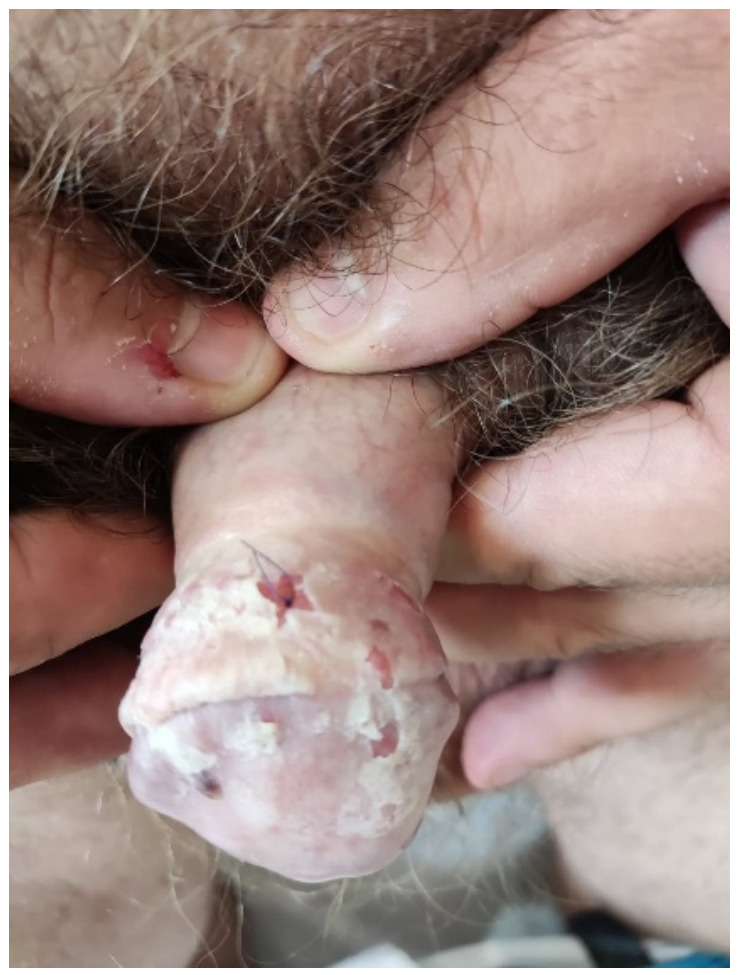
Close-up of glans lesions prior to therapy initiation.

Despite intermittent symptomatic relief, the patient developed progressive hyperkeratosis, fibrotic tightening of the foreskin, recurrent fissuring, and reduced tissue elasticity (Figure 1 and Figure 2). On examination, hyperkeratotic plaques of the glans, areas of atrophic scarring, and a partial constrictive ring of the foreskin were observed. There were no cutaneous, oral, or scalp lesions typical of LP. Given therapeutic resistance and atypical evolution, a diagnostic 4-mm punch biopsy was obtained from an active hyperkeratotic lesion of the glans before any new intervention was considered.

Histopathology demonstrated stratified squamous epithelium with pronounced hyperkeratosis, irregular acanthosis, marked hypergranulosis, prominent basal cell vacuolar degeneration, and a dense band-like lymphohistiocytic infiltrate closely apposed to the dermoepidermal junction-features diagnostic of lichenoid interface dermatitis consistent with lichen planus. No epithelial dysplasia or invasive changes were identified.

## 3. Materials and Methods

### 3.1. Study Design and Ethical Considerations

This is a single-patient, prospective, histopathology-paired interventional case observation conducted in a private dermatologic practice. The patient provided written informed consent for diagnostic procedures, the therapeutic intervention, and publication of anonymized clinical and histopathological data, including clinical photographs. The study was conducted in accordance with the principles of the Declaration of Helsinki and received ethical approval from the Bioethics Committee of the District Medical Chamber in Kraków (L.dz. OIL/KBL/02/2026, 24 February 2026).

### 3.2. Patient Assessment

Disease duration, prior therapies, subjective symptoms (pruritus, burning, fissuring), the macroscopic distribution of hyperkeratotic plaques, and functional impairment were recorded at baseline and at every follow-up visit. Standardized digital photographs were obtained under consistent lighting and positioning. Cutaneous, oral, and appendageal sites typical of LP were inspected and remained free of lesions throughout the observation period.

A detailed treatment and comorbidity history was obtained directly from the patient. Prior to the present intervention, the patient had used several high-potency topical corticosteroid preparations intermittently over the 15-year disease course; one of these preparations was discontinued after approximately one week because of a contact-allergic reaction characterized by marked skin sensitivity resembling a burn, and was replaced with an alternative topical corticosteroid that provided symptomatic benefit but was limited by protocol to a maximum of two weeks of continuous use because of cutaneous atrophy risk. Topical tacrolimus 0.1% had also been trialed previously without a durable benefit. The patient had not used any other topical or systemic medications for this condition and had not undergone any complementary or alternative therapies (e.g., acupuncture) directed at the genital lesions. With respect to comorbidities, the patient reported obesity and a history of genetically elevated triglycerides managed with ezetimibe; no other chronic illnesses and no use of dietary supplements were reported. The patient had not undergone formal testing for sexually transmitted infections at the time of presentation, but reported a long-term monogamous relationship and expressed willingness to undergo such testing if clinically indicated; no clinical findings during the observation period suggested an active sexually transmitted infection. This history is provided to contextualize the refractory nature of the disease and to clarify that no concomitant pharmacological, illicit, or alternative therapies could account for the clinical changes observed during RSCE treatment.

### 3.3. Histopathological Analysis

**Figure 3:**
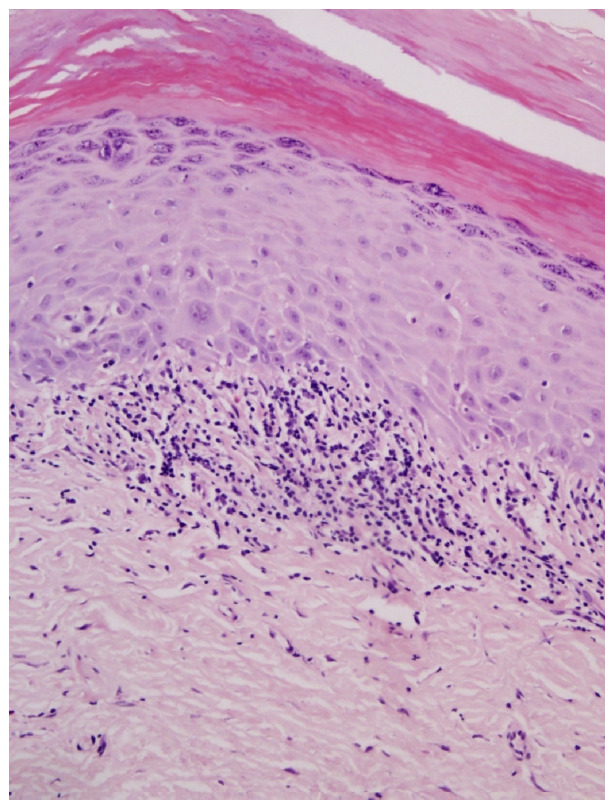
Histopathology before treatment (H&E). Pronounced hyperkeratosis, irregular acanthosis, marked hypergranulosis, basal vacuolar degeneration, and a dense band-like lymphohistiocytic infiltrate at the dermoepidermal junction. Magnification as originally acquired (see Limitations, Section 6).

Punch biopsy specimens (4 mm) were fixed in 10% neutral-buffered formalin for 24 h, processed routinely, embedded in paraffin, and sectioned at 4 µm. Sections were stained with hematoxylin and eosin (H&E) (Figure 3). The follow-up biopsy was obtained four months after initiation of therapy from a previously affected, clinically improved area immediately adjacent to the baseline biopsy site and processed identically. Sections were evaluated qualitatively by a board-certified dermatopathologist (Z.W.) for the following parameters: degree of hyperkeratosis; presence of hypergranulosis; acanthosis; basal cell vacuolar degeneration; density and distribution of the lymphohistiocytic infiltrate; integrity of the dermoepidermal junction; and presence or absence of epithelial dysplasia. Histological evaluation was qualitative; no semi-quantitative, ordinal scoring system was applied, and no immunohistochemistry, in situ hybridization, or transcriptomic analysis was performed. We acknowledge this as a substantial limitation and address it explicitly in Section 6.

### 3.4. Exosome Preparation

The investigational product was a commercial, lyophilized preparation of *Rosa damascena* stem cell-derived exosomes (RSCEs; ExoCoBio Inc., Seoul, Republic of Korea), manufactured using the proprietary ExoSCRT™ mass-culture and purification platform. Each treatment vial contained 20 mg of lyophilized RSCE powder supplied in a dual-container system together with 5 mL of diluent for reconstitution; the solution was prepared ex tempore and stored at 2–8 °C until use. This is the same commercial preparation and batch-release process as that used in a previously published retrospective case series of cutaneous wound-healing and scar-remodeling indications [14]. Regarding regulatory status, the ExoSCRT™ platform output (INCI name: *Rosa damascena* Callus Extracellular Vesicles) is registered as a cosmetic ingredient with the Personal Care Products Council (PCPC) in the United States and is marketed and approved for topical cosmetic use; it is not approved for injection or for use as a medicinal product, and in this case it was applied exclusively topically, in accordance with this regulatory framework. We did not perform independent in-house characterization (e.g., nanoparticle tracking analysis, transmission electron microscopy, Western blot for tetraspanins, or microRNA profiling) of the lot used in this case. Per manufacturer-provided documentation and published compositional studies of this preparation [13,14], the EV size distribution is reported as approximately 90–200 nm round vesicular structures consistent with exosome-like nanovesicle morphology, with proteomic analysis identifying approximately 206 peptides of likely cytosolic and membrane origin and miRNA profiling revealing Let-7 family members (Let-7a, Let-7g, Let-7f), miR-8484, miR-574-5p, and miR-1246, alongside lipid components such as phosphatidylcholine and phosphatidic acid. Each treatment vial contained 20 mg of lyophilized RSCE powder, reconstituted immediately before use with 5 mL of sterile diluent under aseptic conditions and gently agitated until homogeneous.

We explicitly note that, in the absence of independent characterization, the biological identity, particle concentration, and cargo composition of the lot administered to this patient are inferred from manufacturer specifications and prior published data on this product, not measured. This is a substantive limitation and is addressed again in Section 6.

### 3.5. Treatment Protocol

Treatment was initiated immediately after baseline histopathological confirmation of LP. The protocol consisted of biweekly sessions over four months; this duration was predetermined as a fixed observation window for this preliminary report rather than being extended adaptively until a clinical endpoint was reached, and the patient remained on this schedule for the full four months regardless of the rate of improvement observed at intermediate visits. At each session, 2 mL of freshly reconstituted RSCE solution was applied in-office to affected genital areas and gently massaged for uniform distribution. In regions of pronounced hyperkeratosis or fibrosis, superficial microneedling (depth set to superficial dermal penetration) was performed using a sterile disposable device to enhance local delivery. The remaining 3 mL of reconstituted solution was dispensed to the patient for same-day topical application at home in the evening, without occlusion. The patient was instructed to refrain from irritant topicals for 24 h after each session and to maintain standard hygiene.

No concomitant topical corticosteroid, calcineurin inhibitor, or systemic immunomodulator was administered during the treatment period.

### 3.6. Clinical Outcome Assessment

**Figure 4:**
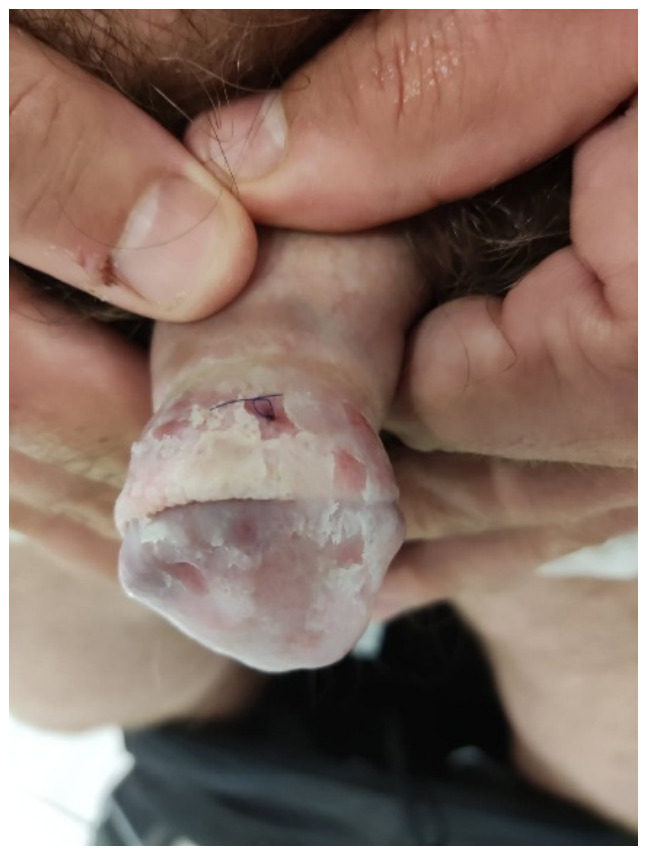
Early desquamation of hyperkeratotic layers during the initial treatment phase.

**Figure 5:**
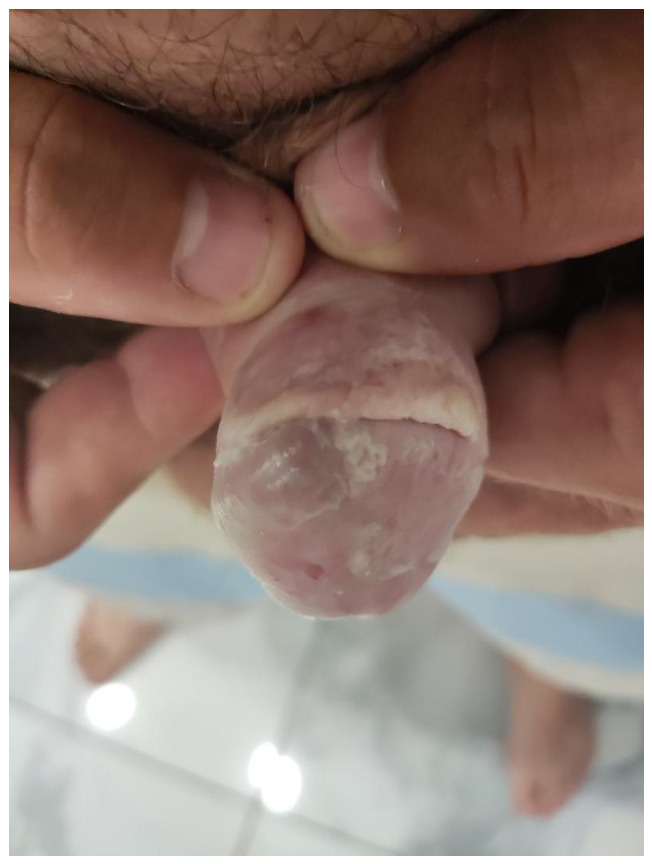
Intermediate clinical improvement with reduction of fibrosis.

**Figure 6:**
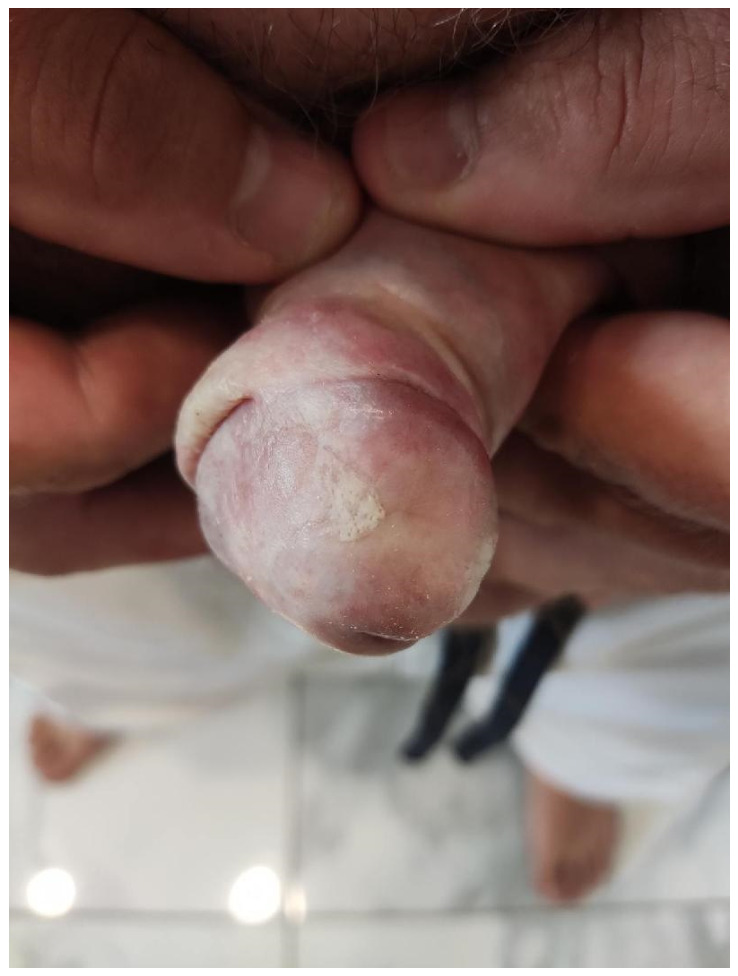
Late clinical outcome after four months, showing normalization of tissue structure.

Clinical response was evaluated at each visit by visual inspection, assessment of hyperkeratosis and fibrosis, evaluation of tissue elasticity, and patient-reported scoring of pruritus, burning, and fissuring. Standardized clinical photography was repeated at consistent intervals (Figure 4, Figure 5 and Figure 6). The primary clinical endpoint was a qualitative improvement in lesion morphology and resolution of subjective symptoms; the primary histopathological endpoint was a qualitative reduction of the lymphohistiocytic infiltrate together with restoration of dermoepidermal interface integrity. No validated patient-reported outcome instrument (e.g., DLQI or a genital-LP-specific instrument) was prospectively administered, which we acknowledge as a limitation.

### 3.7. Safety Monitoring

Adverse events were assessed at every visit, with explicit questioning regarding local irritation, erythema beyond the expected post-procedural response, infection, and systemic symptoms. No systemic laboratory monitoring was performed, given the strictly topical administration.

## 4. Clinical and Histological Outcomes

**Figure 7:**
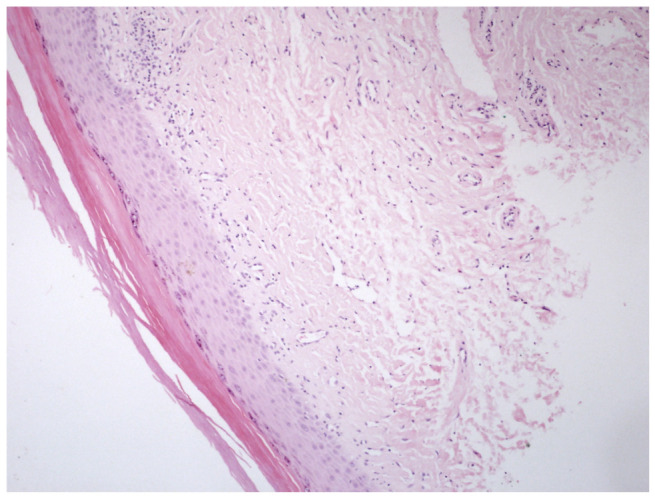
Histopathology after four months of topical RSCE therapy (H&E). Reduced hyperkeratosis and hypergranulosis, attenuation of the lymphohistiocytic infiltrate, and partial restoration of the dermoepidermal interface, with reduced basal vacuolar degeneration. Magnification as originally acquired and not matched to Figure 3; readers are referred to Section 6 for a discussion of this limitation.

Following initiation of RSCE therapy, progressive desquamation of hyperkeratotic layers was observed clinically. Pruritus, burning, and fissuring resolved within the first weeks. Over successive sessions, tissue elasticity improved, and the fibrotic foreskin ring became less pronounced (Figure 7). After four months, the glans surface showed marked normalization without active inflammatory lesions.

**Figure 8:**
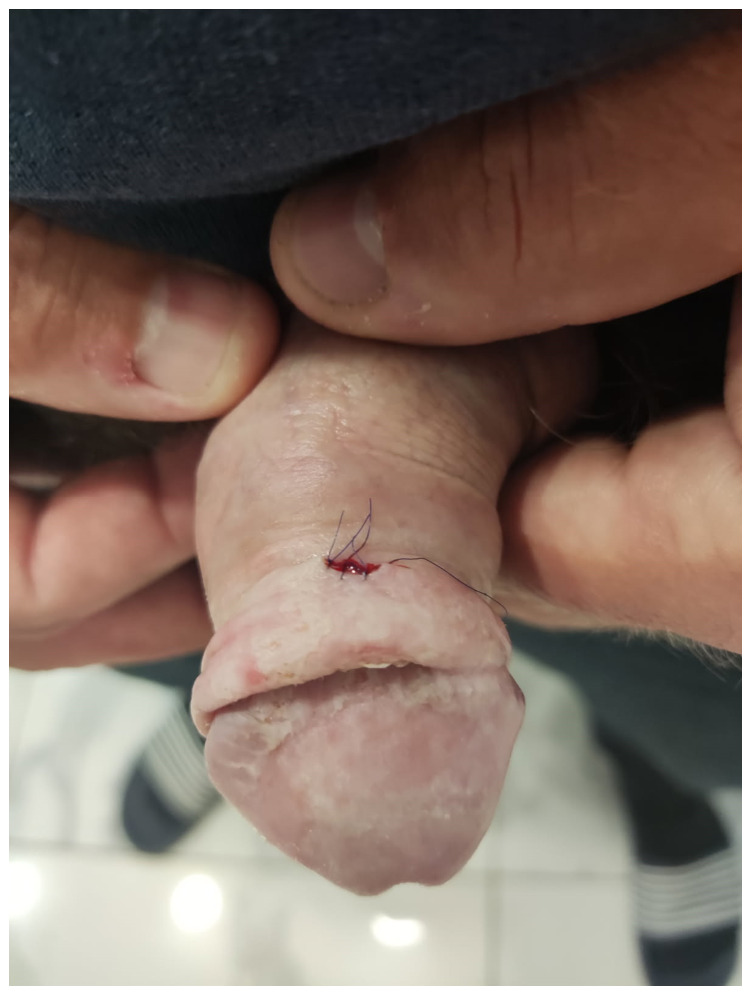
Final clinical appearance at month four, corresponding to the timepoint of the follow-up biopsy shown in Figure 7, demonstrates macroscopic normalization of the glans surface relative to baseline (Figure 2).

Qualitative comparison of the paired biopsies showed reduced hyperkeratosis and hypergranulosis, less prominent acanthosis, attenuation of the band-like lymphohistiocytic infiltrate at the dermoepidermal junction, partial restoration of the dermoepidermal interface, and reduced basal vacuolar degeneration relative to baseline (Figure 3 and Figure 7). No epithelial dysplasia was observed at either time point. We emphasize that this comparison is qualitative and was not supported by ordinal scoring or by immunohistochemistry, and that Figure 3 and Figure 7 are presented at the magnifications at which they were originally acquired (see figure legends), which is a known limitation of the figure presentation in this report. The corresponding macroscopic appearance at the four-month time point is shown in Figure 8.

No local or systemic adverse events were recorded over the four-month treatment period.

## 5. Discussion

In this single case of long-standing male genital LP refractory to high-potency topical corticosteroids and tacrolimus 0.1%, biweekly topical application of a commercial *Rosa damascena* exosome preparation, combined with superficial microneedling, was followed by clinical improvement and qualitative attenuation of the histological hallmarks of interface dermatitis on a paired biopsy taken from an immediately adjacent, previously affected site at four months. The observation that the band-like lymphohistiocytic infiltrate and basal vacuolar degeneration both decreased on paired biopsy, together with restoration of the dermoepidermal interface, is the principal qualitative finding of this report.

We deliberately frame these observations as descriptive and hypothesis-generating. A single uncontrolled case cannot establish causality, cannot isolate the contribution of the exosome preparation from that of microneedling, regression to the mean, or natural fluctuation of chronic LP, and cannot exclude placebo effects on patient-reported outcomes. We therefore avoid quantitative claims of efficacy.

In comparison with conventional therapy, high-potency topical corticosteroids and calcineurin inhibitors achieve symptomatic relief in many patients but rarely produce documented histological normalization in long-standing genital disease [8,9]. JAK inhibitors have shown promise in refractory mucosal LP [10] but act through systemic pathway suppression and carry the risk profile of immunosuppressive therapy. Topical interventions that combine attenuation of local inflammation with support of epithelial integrity, if validated, would be of clinical interest in this anatomical site, where systemic exposure is undesirable and structural recovery is the unmet need.

The mechanism by which plant-derived exosomes might exert any biological effect on interface dermatitis is not established. Published in vitro data on this specific preparation report reduction of IL-6 release by activated macrophages and stimulation of fibroblast proliferation, and a cargo of microRNAs, including Let-7 family members, has been described [13]. From these data alone, however, no specific mechanism can be inferred for the present case. We therefore restrict ourselves to noting that the qualitative histological pattern observed-attenuation of the band-like infiltrate and reduction of basal vacuolar damage with partial restoration of the dermoepidermal junction-is compatible with reduced interface inflammation, and we do not assign this pattern to any specific signaling axis on the basis of the data presented here.

Beyond the vesicle-level characterization summarized above, *Rosa damascena* itself has a long ethnopharmacological record and an increasingly well-described phytochemical profile that may be relevant to the choice of this botanical source for exosome-like nanovesicle production. Across Persian, Middle Eastern, and European traditional medicine systems, *R. damascena* preparations have been used as anti-inflammatory, antiseptic, antispasmodic, and analgesic remedies, and contemporary phytochemical studies have confirmed antimicrobial, antioxidant, anti-inflammatory, and analgesic activity attributable in part to its polyphenol and flavonoid content [14]. The plant material used for ExoSCRT™-based callus culture is therefore derived from a species with a documented bioactive secondary-metabolite profile, in addition to the proteomic and miRNA cargo of the exosome-like vesicles themselves [13,14]. We did not perform independent phytochemical analysis of the callus-derived starting material or of the final RSCE preparation used in this case, and we agree with the reviewer that direct chemical characterization of the bioactive small-molecule and lipid content of this specific product, beyond the vesicle size, protein, and miRNA data already published [13,14], would be a valuable addition to future work on this preparation.

## 6. Limitations

This study has substantial limitations that constrain its interpretation, several of which we are unable to address within this report.

First, this is a single, uncontrolled case. Causality between the RSCE application and the observed clinical and histological changes cannot be established, and natural disease fluctuation, regression to the mean, and the contribution of microneedling itself cannot be excluded.

Second, the histological assessment in this report is qualitative. We did not apply a pre-specified semi-quantitative, ordinal scoring system, and we did not perform blinded re-scoring of the paired biopsies. Outcome assessment also relied on qualitative clinical evaluation; no validated patient-reported outcome instrument was prospectively administered. As a consequence, the histological and clinical comparisons presented are descriptive and not numerically supported.

Third, no immunohistochemical or transcriptomic analyses were performed on the paired biopsy material. Specifically, the inflammatory mediators discussed above (IL-6, TNF-α) and the lymphocytic compartment (CD3, CD4, CD8, FoxP3) were not assessed at the protein or transcript level. We acknowledge this as a major gap; without such data, the histological observations described here cannot be linked to any specific immunological mechanism.

Fourth, Figure 3 and Figure 7 are presented at the magnifications at which they were originally acquired during routine diagnostic histopathology, and these magnifications are not identical between the baseline and post-treatment panels. We were unable to re-photograph the original sections at matched magnifications for this report. We note this explicitly so that readers can interpret the figures with this constraint in mind and recommend that future paired-biopsy reports of this kind use prospectively standardized image acquisition.

Fifth, the exosome preparation used here was a standardized commercial product, but no independent in-house characterization (NTA, TEM, Western blot for tetraspanins, microRNA profiling) of the actual lot administered was performed. The biological identity, particle count, and cargo composition of the administered material are inferred from manufacturer specifications and one external compositional study of this product, not measured.

Sixth, the follow-up window of four months is too short to assess the durability of response or risk of recurrence, and long-term surveillance for malignant transformation in chronic mucosal LP cannot be substituted for by short-term observation.

Adequately powered prospective controlled studies, with independent EV characterization, prospectively standardized blinded scoring, paired biopsies including IHC and ideally transcriptomic profiling, and active comparators (e.g., topical corticosteroids, calcineurin inhibitors, or topical JAK inhibitors), are required before any therapeutic claim can be supported. We hope that the present report, despite its substantial limitations, may motivate such studies.

## 7. Conclusions

In one patient with refractory long-standing male genital lichen planus, biweekly topical application of a commercial *Rosa damascena* stem cell-derived exosome preparation combined with superficial microneedling was associated, over four months, with a clinical resolution of pruritus and fissuring and with qualitative attenuation of the histological hallmarks of interface dermatitis on a paired biopsy from an adjacent site. The histological assessment is qualitative; no immunohistochemical profiling was performed, and the exosome preparation was not independently characterized. We present this observation as hypothesis-generating only. Independent characterization of the exosome preparation, immunohistochemical and ideally transcriptomic profiling of paired tissue, and prospective controlled studies are required before any therapeutic claim can be made.

## Data Availability

All data supporting the findings of this case report are contained within the article. Original H&E slides and FFPE blocks are stored at the Department of Pathology, University of Kalisz, and are available from the corresponding author on reasonable request.
